# MEF2C Expression Is Regulated by the Post-transcriptional Activation of the METTL3-m^6^A-YTHDF1 Axis in Myoblast Differentiation

**DOI:** 10.3389/fvets.2022.900924

**Published:** 2022-04-28

**Authors:** Xinran Yang, Yue Ning, Sayed Haidar Abbas Raza, Chugang Mei, Linsen Zan

**Affiliations:** ^1^College of Animal Science and Technology, Northwest A&F University, Xianyang, China; ^2^College of Chemistry and Chemical Engineering, Xianyang Normal University, Xianyang, China; ^3^National Beef Cattle Improvement Center, Northwest A&F University, Xianyang, China

**Keywords:** N^6^-methyladenosine, myoblast differentiation, MEF2C, METTL3, cattle

## Abstract

*N*^6^-methyladenosine (m^6^A) plays an essential role in regulating gene expression. However, the effect of m^6^A on skeletal myoblast differentiation and the underlying mechanisms are still unclear. Here, we ascertained mRNA m^6^A methylation exhibited declined changes during bovine skeletal myoblast differentiation, and both *MEF2C* mRNA expression and m^6^A levels were significantly increased during myoblast differentiation. We found that *MEF2C* with mutated m^6^A sites significantly inhibited myoblast differentiation compared with wild-type *MEF2C*. METTL3 promoted MEF2C protein expression through posttranscriptional modification in an m^6^A-YTHDF1-dependent manner. Moreover, MEF2C promoted the expression of METTL3 by binding to its promoter. These results revealed that there is a positive feedback loop between these molecules in myoblast differentiation. Our study provided new insights into skeletal muscle differentiation and fusion, which may provide an RNA methylation-based approach for molecular genetics and breeding in livestock as well as for the treatment of muscle-related diseases.

## Introduction

Research on the mechanisms underlying the growth, development and regeneration of skeletal muscle is of great significance to livestock meat production and meat quality improvements (1). The growth and development of skeletal muscle are extremely complex biological processes, which successively include directional differentiation of progenitor cells, myoblast proliferation, differentiation, and fusion of myocytes, and finally, formation of multinucleated muscle fibers with contractile function (2). Epigenetic changes such as DNA methylation and histone methylation, in addition to some myogenic-specific transcription factors, are known to play a key role in skeletal myogenesis (3, 4). Nonetheless, molecular selection breeding in beef cattle mostly focused on the exploration of some key genes, and rarely improved the breeding process from the perspective of RNA.

RNA methylation accounted for more than 60% of all identified chemical RNA modifications (5). Among these types of modification, *N*^6^-methyladenosine (m^6^A) is considered the most common mRNA modification in eukaryotes (6–12). m^6^A is a dynamic and reversible posttranscriptional methylation modification, that is catalyzed by m^6^A writer proteins (methyltransferase complexes composed of METTL3, METTL14, and WTAP) and is demethylated by m^6^A eraser proteins (FTO and ALKBH5) (13–17). The m^6^A modification is functionally interpreted by m^6^A “reader” proteins, such as the widely studied YTH-domain family proteins (18, 19). m^6^A modification plays a variety of roles in mRNA metabolism, including mRNA translation efficiency, stability and splicing (19–23). A growing body of evidence suggested that m^6^A influences a variety of biological processes, such as multiple cancer processes, mESC differentiation, antitumor immunity, cell fate determination, and adipogenesis (23–27). A series of recent studies, including ours, have published transcriptome profiles of m^6^A modifications in muscle development in farm animals such as pigs, cattle, sheep and geese, revealing the important role of m^6^A methylation modifications in skeletal muscle growth and development (28–36). However, the m^6^A levels in the process of skeletal muscle myoblast differentiation and the detailed molecular mechanism underlying its role in this process are still unclear. Moreover, there are few reports on the biological functions of m^6^A modification in cattle.

In the present study, we investigated the abundance, function and mechanism of m^6^A modifications during myogenic differentiation in bovine myoblast. First, we found significant changes in the levels of m^6^A modification in the mRNA of myoblasts and myotubes. We screened and identified the mechanism by which MEF2C expression was regulated by the METTL3-m^6^A-YTHDF1 axis, and these findings were verified by MeRIP-seq, RNA-seq, and experiments. Then, it was found that MEF2C promotes the expression of METTL3 by directly binding to its promoter region. Our study identified the interaction of MEF2C and m^6^A modification via a feedback loop during bovine skeletal muscle differentiation *in vitro*. Our findings make the mechanism of m^6^A modification in skeletal myogenesis increasingly clear and provide an important basis for improving the molecular breeding of livestock from the perspective of RNA modification.

## Materials and Methods

### Culture and Differentiation of Bovine Myoblasts

The cells used in this study were skeletal myoblasts of Qinchuan beef cattle preserved in our laboratory (36, 37). The myoblasts were cultured to 80–90% confluence in growth medium (GM), and then, myogenic differentiation was induced with differentiation medium (DM). The culture conditions were a humidified incubator (Thermo Fisher Scientific, MA, USA) containing 5% carbon dioxide at 37°C. The myoblast growth medium was composed of DMEM/F12 (HyClone, UT, USA), 20% fetal bovine serum (FBS, GIBCO, NY, USA) and 1% penicillin/streptomycin. The myoblast differentiation medium consisted of DMEM/F12 containing 2% horse serum (HS, GIBCO) and 1% penicillin/streptomycin. The medium was changed every 2 days.

### RNA Isolation, CDNA Synthesis, and Real-Time Fluorescence Quantitative PCR (RT-QPCR)

RNAiso reagent (Takara, Dalian, China) was used to lyse proliferating myoblasts (named GM; 90% confluence, cultured in GM) and differentiated myotubes (named DM; cultured in DM for 4 d), and isolate the total RNA. PrimeScript RT reagent kit (Takara) was used to synthesize cDNA. The residual genomic DNA was removed at 42°C for 2 min, and then, the reverse transcription reaction was conducted at 37°C for 15 min and then at 85°C for 5 s. RT-qPCR was performed using the TB Green Premix Ex Taq II Kit (Takara) in the CFX Connect qPCR Detection System (BIO-RAD, CA, USA). Bovine *GAPDH* was used as the internal reference to standardize the data. Each sample analyzed by RT-qPCR was subjected to at least three biological repeats. Primers used for RT-qPCR are listed in Supplementary Table 1. Relative mRNA expression was calculated using the 2^−Δ*ΔCt*^ method (38).

### Analysis of the m^6^A Levels in MRNA Using LC-MS/MS

Total RNA was purified using a PolyATtract mRNA Isolation Systems kit (Promega, WI, USA) following the manufacturer's protocols. About 200 ng of mRNA was digested in 25 μl buffer with nuclease P1 (2U; M0660S, NEB, MA, USA), 2.5 mM ZnCl_2_ and 25 mM NaCl at 42°C for 2 h. Then alkaline phosphatase (0.5 U; Thermo Fisher, MA, USA) and NH_4_HCO_3_ (1 M, 3 ml) were added and incubated at 37°C for 2 h. Next, the sample was diluted 5-fold and filtered (pore size 0.22 mm; MilliporeSigma, MA, USA). A total of 5 μl of the solution was injected into an LC-MS/MS instrument. The total amount of m^6^A in mRNA was assessed using an Agilent 1290 Infinity II-6470 (Agilent Technology, CA, USA) system as previously reported (15). The concentration of m^6^A and A was calculated according to the standard curve, and then m^6^A/A (%) was obtained.

### Dot Blotting

The purified mRNA was diluted to to a concentration of 100 ~ 200 ng/μl, denatured at 95°C for 3 min, and immediately put on ice. Approximately 1 μl mRNA or total RNA was spotted on a Hybond-N^+^ membrane (GE Healthcarea, IL, USA) and UV-crosslinked for 15 min. After blocking with 5% non-fat milk for 2 h, the membranes were incubated with anti-m^6^A antibody (202003, Synaptic Systems, Germany) overnight at 4°C. Then, the membranes and secondary antibodies incubation 2 h at room temperature. The membranes were imaged using the chemiluminescence method and the ChemiDoc XRS+ Imaging System (BIO-RAD, CA, USA). The developed membranes were stained with 0.02% methylene blue (MB) for 30 min to ensure equal of RNA loading.

### Plasmid Construction, RNA Interference and Transfection

The coding sequences (CDS) of bovine *FTO* (NM_001098142), *METTL3* (NM_001102238), and *YTHDF1* (NM_001191416) were synthesized via PCR and cloned into the pcDNA3.1 expression plasmid. The bovine *MEF2C* (ENSBTAT00000086206.1) CDS was synthesized and cloned into the pcDNA3.1-3xFlag-EGFP-C expression plasmid to generate the wild-type construct (*MEF2C*-WT). Nucleotide 1248 of the *MEF2C* CDS within the m^6^A consensus sites was mutated from adenosine to cytosine (5′-GGACT-3′ → 5′-GGCCT-3′), and this sequence was synthesized by Sangon Biotech (Shanghai, China). All the primers are listed in Supplementary Table 1. Genepharma (Shanghai, China) synthesized all siRNAs for this study, and the sequences are shown in Supplementary Table 2.

To construct the wild-type *MEF2C* reporter plasmid, the entire sequence of *MEF2C* coding region was cloned into a psiCHECK2 vector (Promega) carrying Renilla luciferase and Firefly luciferase. Simultaneously, the adenosine (A) bases in the predicted m^6^A common site were replaced with cytosine (C) bases to generate the *MEF2C* mutant reporter plasmid. Moreover, the amplified fragments of the *METTL3* promoter region containing the wild-type or mutated MEF2C-binding site were subcloned into a pGL3-promoter vector (Promega).

When the myoblasts reached 80-90% confluence, the cells were seeded in 6-well plates. The instantaneous transfection procedure was performed according to the protocol of the Lipofectamine 3000 transfection reagent (Invitrogen, CA, USA), and three replicate wells were transfected each time.

### MRNA Stability Measurement

The cells were treated with actinomycin D (5 μg/ml, Selleck Chemicals, TX, USA) for 0, 3, 6, and 9 h before collection. Then total RNA was extracted, cDNA was synthesized, and the mRNA level was detected by RT-qPCR.

### MeRIP-QPCR of Target Genes

m^6^A immunoprecipitation assays were performed as previously described (39). In brief, 48 h after transfection, RNA from the cells was chemically digested into 200-nt fragments, and more than 200 μg of total RNA was subjected to immunoprecipitation with affinity-purified m^6^A-specific antibody (202003, Synaptic Systems). The RNA fragments that bound to m^6^A were separated by TRIzol reagent. Then the Input RNA and IP RNA were reverse transcribed, and the enriched sequences were detected by RT-qPCR. The ΔΔCt between the 10% input and the IP RNA was measured, and the relative fold change was calculated as 2^−Δ*ΔCt*^. Supplementary Table 1 lists the primers for amplification of the m^6^A peak sequences.

### Western Blotting

Cells were collected and lysed on ice in Western and IP cell lysis buffer (Beyotime Biotech, Shanghai, China) containing 1% PMSF (Solabio Life Sciences, Beijing, China) and 10% phosphatase inhibitor cocktail (Roche, Germany) for 30 min. The lysates were collected with a cell scraper and centrifuged at 14,000 g for 10 min at 4°C to collect the proteins in the supernatants. Then, the protein concentrations were determined by a BCA protein analysis kit (Beyotime Biotechnology). All the cell proteins were incubated at 100°C for 10 min in SDS-PAGE sample buffer. The proteins were separated by SDS-PAGE and transferred to PVDF membranes for immunoblotting. The membranes were incubated overnight with primary antibodies at 4°C and then with secondary antibodies at room temperature for 1.5 h. Western blotting was performed using the chemiluminescence method (ECL Plus detection system) and the band intensities were quantified by ImageJ (NIH, MD, USA) software. The antibodies are listed in Supplementary Table 3.

### Immunofluorescence

Cultured myoblasts and myotubes were washed briefly with PBS and fixed with 4% paraformaldehyde at room temperature for 20 min, and then permeabilized with PBS containing 0.5% Triton X-100 for 15 min. The cells were subsequently washed three times with PBS. The cells were blocked with 0.3 M glycine, 10% donkey serum and 1% BSA in PBS for 1 h. Then, the cells were incubated with primary antibodies overnight at 4°C. After washing 3 times with PBS, the cells were incubated with fluorescent dye-conjugated secondary antibodies for 1.5 h at room temperature, and this step was performed in the dark. The cells were washed 3 times with PBS, stained with 0.1% DAPI (Sigma-Aldrich, MO, USA) for 15 min and then visualized under a fluorescence microscope [Olympus IX71 (Olympus Corporation, Japan) or Evos-fl-auto2 fluorescence microscopy imaging system (Thermo Fisher)]. The antibodies are listed in Supplementary Table 3.

### Luciferase Reporter Assay

293A cells were inoculated in 24-well plates and transfected with a luciferase reporter, the pRL-TK Renilla luciferase vector (Promega), and the *METTL3* or *MEF2C* expression vectors. At least three biological replicates were set for each group. The relative luciferase activities were measured 48 h after transfection using a Dual-Luciferase Reporter Assay Kit (Promega).

### RNA Immunoprecipitation Assay

RIP was performed as previously described (19). Bovine skeletal myoblasts transfected with pcDNA3.1-*YTHDF1* or the negative control were collected with cell scrapers (two 15-cm culture dishes for each group). Cell lysis and RNA immunoprecipitation and purification were performed according to the protocol of the Magna RIP RNA-Binding Protein Immunoprecipitation Kit (Sigma-Aldrich). The input mRNA and IP RNA of each sample were reverse transcribed, and enrichment was determined by RT-qPCR.

### Chromatin Immunoprecipitation Assay

ChIP was performed using SimpleChIP Plus Sonication Chromatin IP Kit (Cell Signaling Technology, MA, USA) following the protocol of the manufacturer. In short, bovine skeletal myoblasts transfected with MEF2C expression vectors or empty vectors were fixed with 1% formaldehyde and then Quenching buffer was added to terminate the crosslinking reaction. The chromatin was sheared using a Covaris M220 focused-ultrasonicator (Woburn, MA, USA). The chromatin was incubated with either an anti-MEF2C polyclonal antibody (10056-1-AP, Proteintech, Wuhan, China) or an anti-IgG overnight at 4°C, and the IPs were bound to Protein G magnetic beads. Next, the chromatin was eluted from the antibody/protein G beads and de-crosslinked. The purified DNA and enriched DNA sequences were detected using RT-qPCR with the primers listed in Supplementary Table 1. The data were expressed as a percentage of input.

### Statistical Analysis

All results were displayed as the means ± standard deviation (SD) of at least three biological repetitions. Student's *t*-test (between two groups) or ANOVA (among multiple groups) were used to compare the significance of the means. *p* < 0.01 or *p* < 0.05 were considered the differences to be very significant or significant, respectively. GraphPad Prism 7.00 (GraphPad Software, CA, USA) software was used to analyze the results and produce images.

## Results

### Identification of Bovine Skeletal Myoblasts and Detection of m^6^A Levels During Differentiation

**Figure 1:**
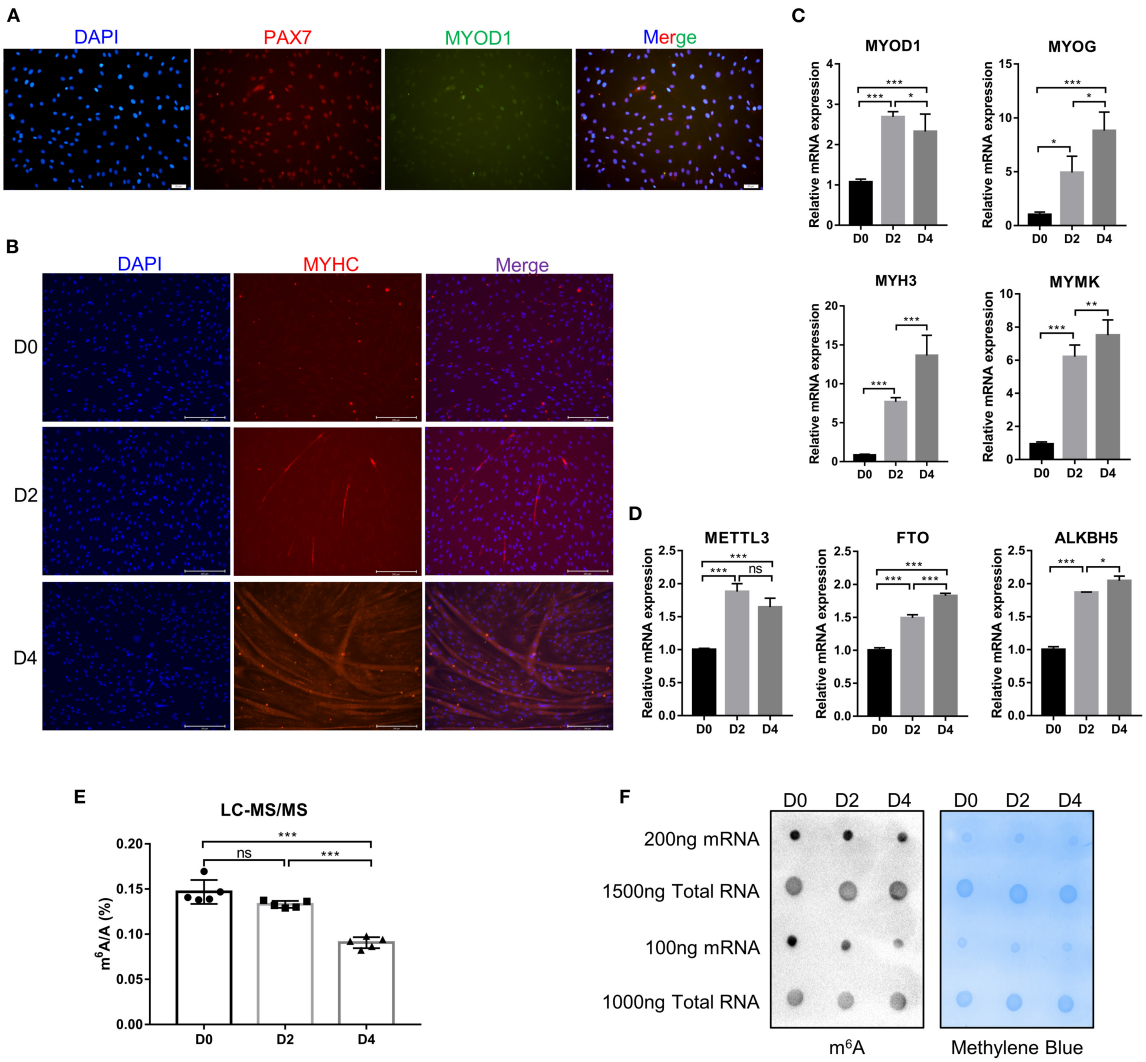
Identification of bovine skeletal myoblasts and detection of m^6^A levels during myogenic differentiation. **(A)** Identification of myoblasts based on PAX7 and MYOD1 expression in GM (scale bar: 50 μm, Olympus IX71). **(B)** Identification of differentiated myotubes based on MYHC expression after culture in DM for 0, 2, and 4 days (scale bar: 200 μm, Evos-fl-auto2 microscopy imaging system). **(C)** Relative mRNA expression of myogenic genes (*MYOD1, MYOG, MYH3*, and *MYMK)* during bovine skeletal myoblast differentiation. **(D)** Relative mRNA expression of *METTL3, FTO* and *ALKBH5* during myoblast differentiation. The results are presented as the means ± SD. **(E)** LC-MS/MS assay showed the amount of mRNA m^6^A during myoblast differentiation. Data are presented as mean ± SD (*n* = 5). **(F)** Dot blotting was used to detect the m^6^A modification of mRNA and total RNA at D0, D2, and D4 during bovine skeletal myoblasts differentiation. Methylene blue (MB) staining was used as a loading control. **(C–E)** *p < 0.05, **p < 0.01, ***p < 0.001, ns, no significance.

Myogenic differentiation and myogenesis are complex biological process. To verify whether the cells isolated from bovine longissimus dorsi muscles can undergo myogenic differentiation, we seeded the isolated cells in culture dishes, grew them to 80–90% confluence, and passaged them to a 6-well plate for further culture. After 48 h in growth medium, immunofluorescence showed that PAX7 and MYOD1 were simultaneously expressed, while the expression of MYOD1 was relatively low (Figure 1A), so we preliminarily identified the isolated cells as myoblasts. The myoblasts were cultured in growth medium to 90% confluence, and induce myogenic differentiation was induced with differentiation medium. Microscopic observation and fluorescence detection of the MYHC protein revealed that the myotubes formed by myoblast fusion gradually increased and became longer as the number of days of differentiation increased (Figure 1B). We evaluated the differentiation status of the myoblasts by detecting the mRNA levels of *MYOD1, MYOG, MYH3* (myosin heavy chain 3), and *MYMK* (myomaker, myoblast fusion factor), which are widely recognized marker genes of differentiated myoblasts and fused myotubes (40). The levels of *MYOG, MYH3*, and *MYMK* gradually increased during myogenic differentiation, while the levels of *MYOD1* peaked on D2 (Figure 1C). These findings were consistent with previous studies showing that MyoD1 plays a vital role in the proliferation and early differentiation of myoblasts (41). The trends in the expression of these pivotal genes were consistent with the differentiation stage. These results suggested that the isolated bovine skeletal myoblasts could undergo myogenic differentiation, and these cells can be used as a model for our follow-up study on myogenic differentiation. Meanwhile, we found the mRNA expression of *FTO* and *ALKBH5* increased during bovine myoblasts differentiation, while the levels of *METTL3* peaked on D2 (Figure 1D). Furthermore, using an LC-MS/MS assay, we detected the m^6^A levels on days 0, 2, and 4 of myogenic differentiation (Figure 1E, Supplementary Figures 1A,B) and found that the levels of m^6^A on D4 were significantly lower than those before differentiation (D0). The result may be related to the increased expression of *FTO* and *ALKBH5*. Alternatively, elevated *METTL3* expression did not lead to the increase of m^6^A level in myoblast differentiation, implying that the regulation of m^6^A modification in bovine myoblasts may be complex. The results of dot blotting also confirmed these same results (Figure 1F). Interestingly, dot blotting analysis showed that there was no obvious difference in the levels of m^6^A on the total RNA (Figure 1F). These results indicated that m^6^A modification may play a potential role in skeletal myogenic differentiation.

### MeRIP-Seq and RNA-Seq Identify Potential Targets of m^6^A Modification in Bovine Myoblasts Differentiation

**Figure 2:**
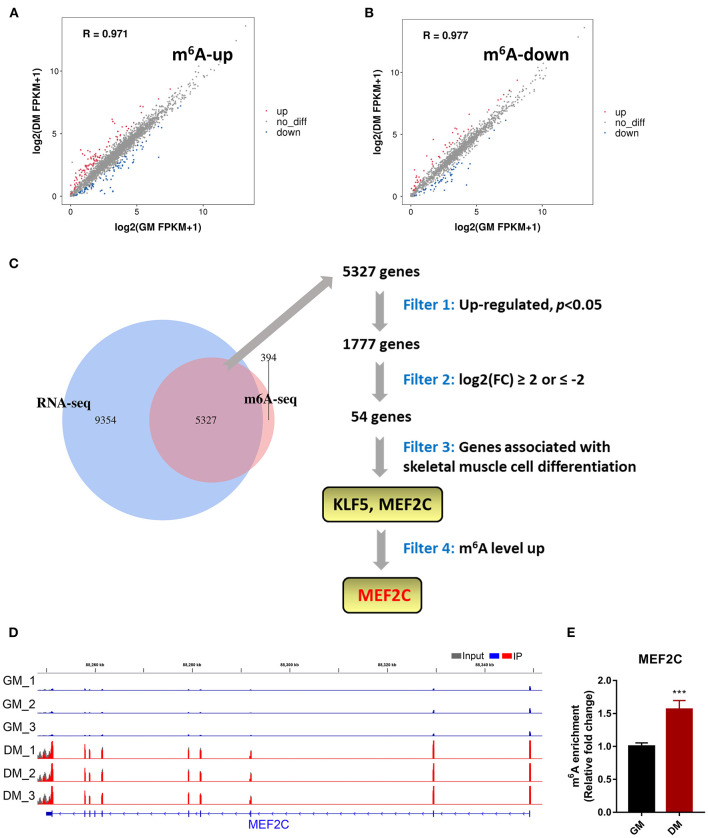
m^6^A methylome profiles of bovine skeletal muscle myoblasts (GM) and myotubes (DM). **(A,B)** Distribution of genes with significant differences in the both m^6^A level and gene expression level between the GM and DM. **(C)** Venn diagram showed the overlapping genes identified by MeRIP-seq and RNA-seq, and bioinformatic analysis identified MEF2C as a target gene of m^6^A modification during bovine skeletal myoblast differentiation. **(D)** IGV tracks displayed the distribution of m^6^A peaks in the MEF2C transcript in all 6 groups. **(E)** Validations of the m^6^A enrichment of MEF2C mRNA by MeRIP-qPCR. Data were presented as means ± SD. ***p < 0.001; Student's *t*-test.

To determine the role of m^6^A in the process of myoblast differentiation, we extracted mRNA from pre-differentiation (GM, myoblasts, D0) and post-differentiation (DM, myotubes, D4) cells for MeRIP-seq and RNA-seq analyses (36). m^6^A abundance has been reported to affect mRNA levels (9, 19, 42). To evaluate whether there was a potential correlation between m^6^A mRNA methylation and gene transcript levels during myoblast differentiation, we compared the differentially expressed genes (DEGs) with the list of transcripts with altered m^6^A levels. On the one hand, we found that among the 3,891 transcripts with higher m^6^A levels in the DM group than in the GM group, only 119 transcripts showed higher expression levels while 94 transcripts exhibited lower expression levels (Figure 2A, Supplementary Table 4). On the other hand, among the 1,751 transcripts whose m^6^A levels were decreased in the DM group compared to the GM group, 58 and 55 transcripts exhibited higher and lower expression levels, respectively (Figure 2B, Supplementary Table 5). The results suggested that the regulation of m^6^A modification on gene expression during bovine myoblast differentiation may be complex. Gene overlap analysis in RNA-seq and MeRIP-seq data showed that 5,327 genes were modified with m^6^A (Figure 2C). Among these 5,327 genes, the expression of 1,777 was upregulated. The skeletal muscle formation is known to be accompanied by the expression of many regulatory factors (43). Therefore, the genes with upregulated mRNA expression during myogenic differentiation were likely potential targets. Two genes, *KLF5* and *MEF2C*, that exhibited significantly increased expression in DM [*p* < 0.05, log_2_(FC) ≥ 2] and are related to skeletal muscle cell differentiation were selected as potential candidates (Figure 2C). Finally, we selected *MEF2C* with increased m^6^A level, as the target for our follow-up molecular mechanism research. Significant differences in the m^6^A peaks were observed in MEF2C, as shown by IGV software (Figure 2D). MeRIP-qPCR was then used to verify the difference in the m^6^A levels of *MEF2C* mRNA between the GM and DM groups. As expected, the m^6^A level of *MEF2C* mRNA exhibited a significant increase in the DM group after myogenic differentiation (Figure 2E).

### MEF2C Promotes the Myogenic Differentiation of Bovine Skeletal Myoblast

**Figure 3:**
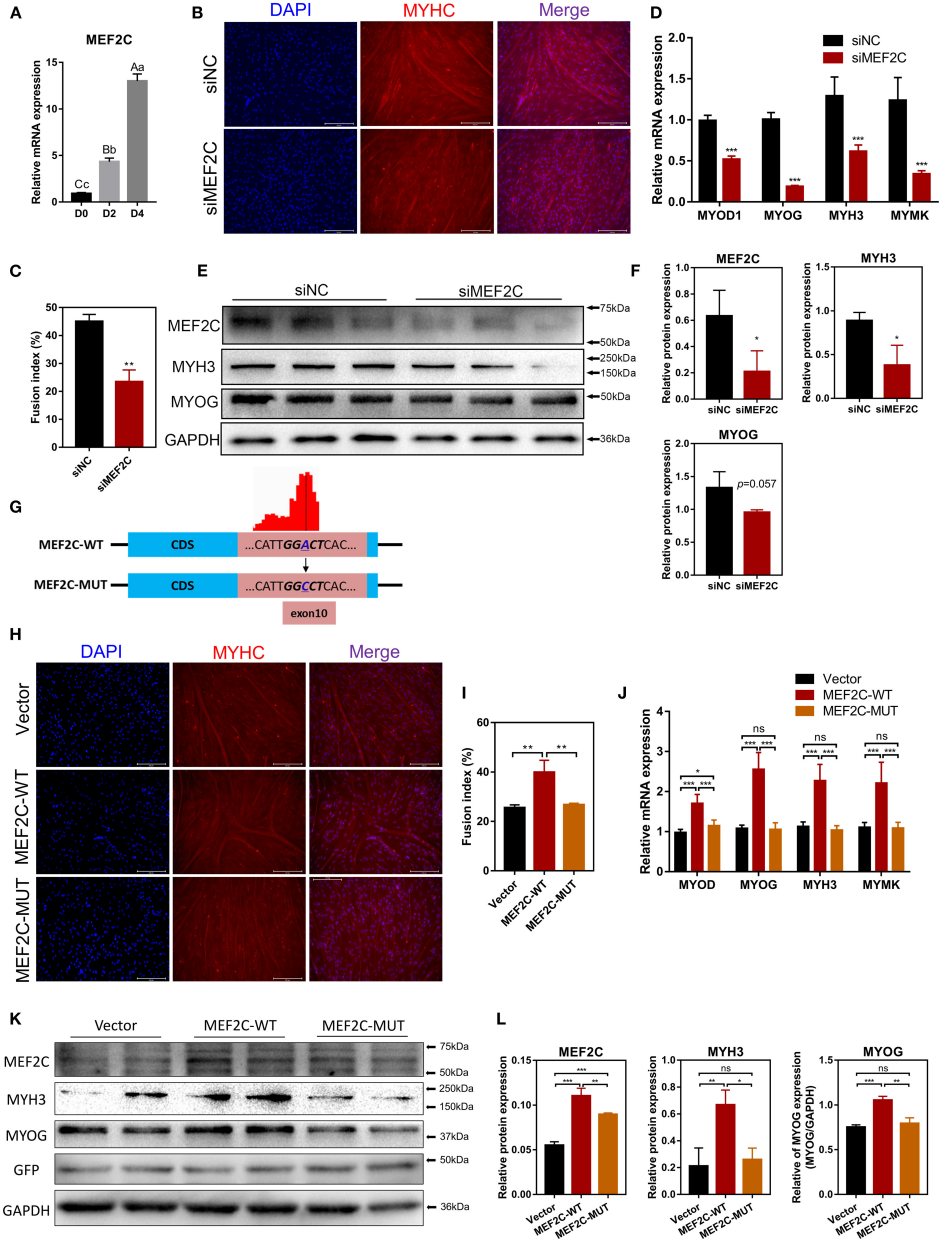
MEF2C promotes bovine skeletal myoblast differentiation, and its expression and function in myoblast differentiation are regulated by m^6^A modification. **(A)** Relative mRNA expression of *MEF2C* during bovine skeletal myoblast differentiation. **(B–F)** After myoblasts were transfected with siRNA for 24 h, myogenic differentiation was induced. **(B)** Myotube formation was observed on the 4th day of differentiation (scale bar: 200 μm). **(C)** The fusion index was calculated as the percentage of nuclei in fused myotubes out of the total nuclei. **(D)** Relative mRNA expression levels of *MYOD1, MYOG, MYH3*, and *MYMK* were measured on the fourth day of differentiation. **(E)** The protein expression of MEF2C, MYH3, MYOG, and GAPDH on the 4th day after myogenic induction. **(F)** Semi-quantitative analysis of the protein expression. **(G)** Synonymous mutations at the m^6^A motif in the MEF2C CDS. **(H–L)** After transfection of the empty control, MEF2C-WT and MEF2C-MUT plasmids for 24 h, myoblasts were induced to differentiate. **(H)** Myotubes with MYHC expression on the 4th day after differentiation (scale bar: 200 μm). **(I)** The fusion index was counted. **(J)** Relative mRNA expression levels of *MYOD1, MYOG, MYH3*, and *MYMK* were measured. **(K,L)** The protein expression of MEF2C, MYH3, MYOG, GFP, and GAPDH after myogenic induction for 4 days. **(D,F,J,L)** The results were normalized to GAPDH levels and are presented as the means ± SD. *p < 0.05, **p < 0.01, ***p < 0.001, ns, no significance.

MEF2C has been reported to play an indispensable role in skeletal myogenesis and myoblast differentiation (44, 45). The expression of *MEF2C* was significantly increased in myoblast differentiation (Figure 3A). To verify the effects of MEF2C on bovine skeletal myoblast differentiation, siMEF2C for loss-of-function assays and *MEF2C*-WT for MEF2C overexpression were synthesized and constructed (Supplementary Figure 2A). MEF2C knockdown apparently inhibited myotube formation and fusion index at day 4 of myogenic differentiation in myoblast (Figures 3B,C). Meanwhile, MEF2C knockdown suppressed the mRNA expression of skeletal muscle-specific myogenic factors, such as *MYOD1, MYOG, MYH3*, and *MYMK*, and decreased the protein expression of MYH3 and MYOG (Figures 3D–F). In contrary, the overexpression of MEF2C obviously increased the mRNA and protein expression of these genes, and promoted myotubes formation and fusion index (Figures 3H–L, Vector and *MEF2C*-WT groups). Taken together, these results demonstrated that MEF2C plays a positive role in myogenic differentiation, which was consistent with expectations.

### m^6^A Modification Facilitates MEF2C Protein Expression

In this study, we confirmed that the expression of *MEF2C* upregulated during myogenic differentiation, and the level of *MEF2C* mRNA m^6^A modification was also significantly increased. Therefore, we speculated that m^6^A modification was involved in the regulation of MEF2C expression. Considering the MeRIP-seq data and the m^6^A peak map of *MEF2C*, we found that the m^6^A-modified motif in *MEF2C* mRNA was in exon 10 (Figures 2D, 3G). Subsequently, we replaced the *N*^6^-methylated adenosine (A) in the m^6^A peaks sequence of *MEF2C* mRNA (exon 10 of the CDS) with cytosine (C) to establish a synonymous mutant *MEF2C* that cannot be modified by m^6^A (Figure 3G). Figures 3H,I show that *MEF2C*-MUT significantly inhibited the *MEF2C*-WT-induced myotube formation. Consistently, compared with *MEF2C*-WT, *MEF2C*-MUT markedly reduced the mRNA expression of *MYOD1, MYOG, MYH3*, and *MYMK* and decreased the protein expression of MYH3 and MYOG, but the mRNA expression of *MYOD1* in the *MEF2C*-MUT group was still higher than that of the control group (Figures 3J–L). It should be noted that *MEF2C*-MUT significantly increased the protein expression of MEF2C compared with the control, but decreased the protein expression of MEF2C compared with *MEF2C*-WT (Figures 3K,L). These results indicate that synonymous mutation of the m^6^A motif in the *MEF2C* CDS inhibits the protein expression of MEF2C.

**Figure 4:**
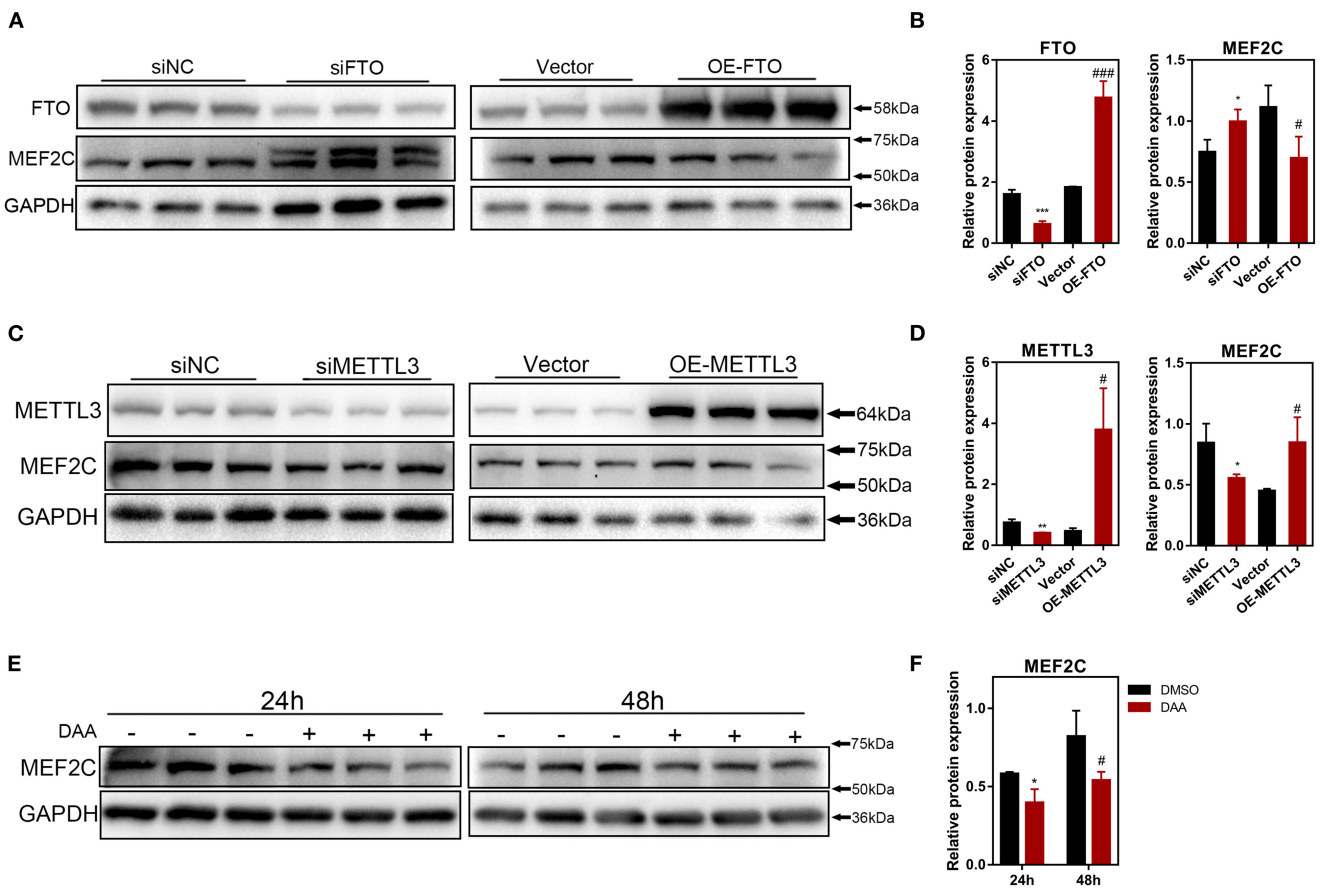
The protein expression of MEF2C is regulated by m6A modification. **(A,B)** MEF2C and FTO protein levels after FTO knockdown and overexpression on the 4th day after differentiation. **(C,D)** MEF2C and METTL3 protein levels after METTL3 knockdown and overexpression for 4th day post differentiation. **(E,F)** Treatment with a global methylation inhibitor, 3-Deazaadenosine (DAA), for 24 and 48 h led to the downregulation of the MEF2C protein levels in bovine skeletal myoblasts. The results are presented as the means ± SD (*p < 0.05, **p < 0.01, ***p < 0.001, siNC vs. siRNA samples and DMSO vs. DAA at 24h; ^#^*p* < 0.05, ^#^^#^^#^*p* < 0.001, empty vector vs. overexpression plasmid samples and DMSO vs. DAA at 48h), using Student's *t* test.

To further investigate how m^6^A methylation modification regulates the expression of MEF2C, we performed loss-of-function and gain-of-function assays with both m^6^A methyltransferase METTL3 and m^6^A demethylase FTO (Supplementary Figures 2B–D). Myoblasts with FTO expression knocked down demonstrated significant upregulation of MEF2C protein expression, whereas FTO overexpression in myoblasts resulted in a reduction in the MEF2C protein levels (Figures 4A,B). In contrary, METTL3 knockdown inhibited the protein expression of MEF2C, while overexpression of METTL3 promoted the protein expression of MEF2C (Figures 4C,D). Additionally, MEF2C protein level was substantially decreased after 24 and 48 h of treatment with a global methylation inhibitor, 3-deazaadenosine (DAA) (4 μM, 86583-19-9, TargetMOI, MA, USA), without affecting its mRNA expression (Figures 4E,F, Supplementary Figure 2E). Together, these findings suggest that the m^6^A modification of mRNA regulates MEF2C gene expression post-transcriptionally.

### METTL3 Upregulates the Protein Expression of MEF2C in an m^6^A-YTHDF1-Dependent Manner

**Figure 5:**
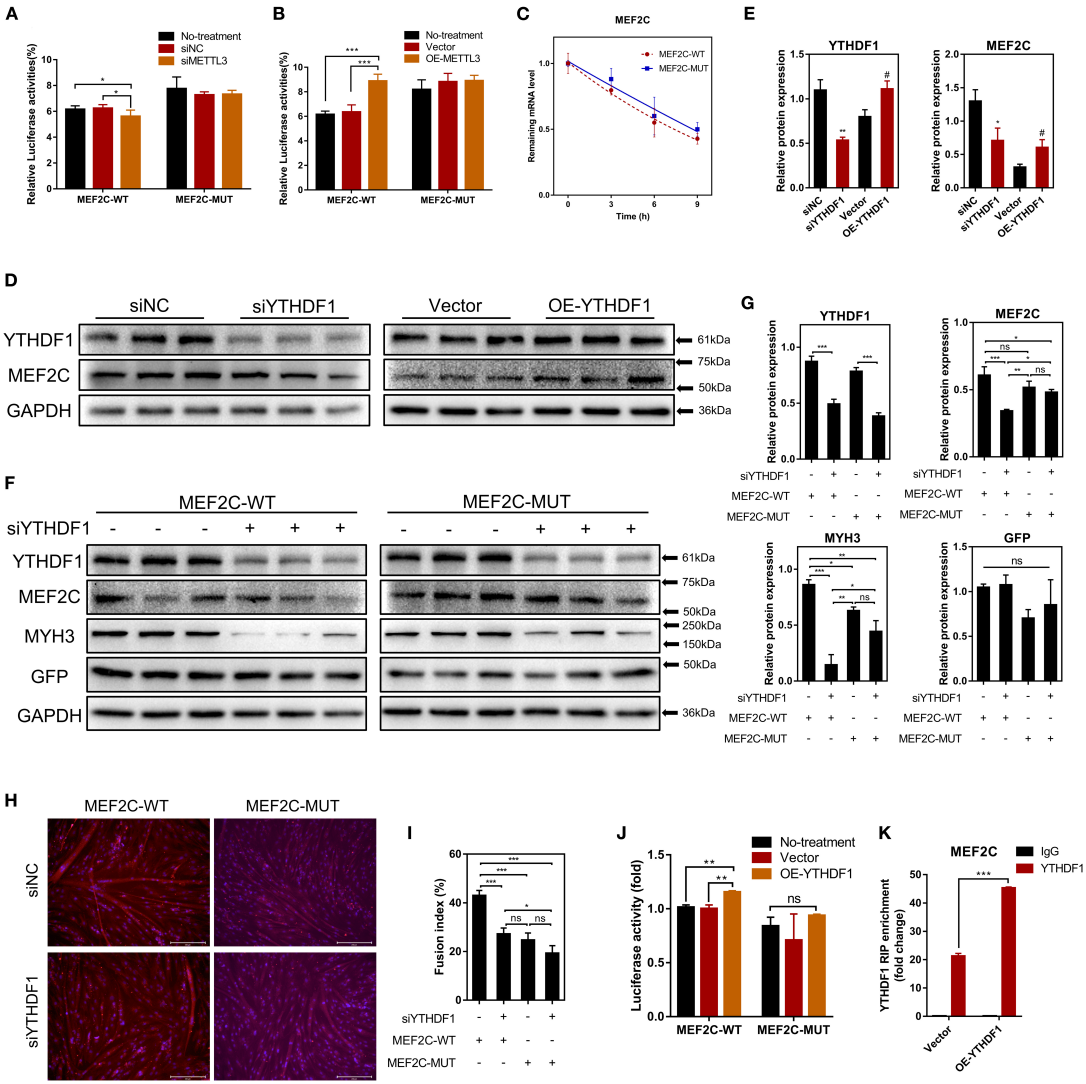
METTL3 increases MEF2C protein expression in an m^6^A-YTHDF1-dependent manner. **(A,B)** Relative dual-luciferase reporter activity of WT (MEF2C-WT) or mutated (MEF2C-MUT) reporters in METTL3 knockdown or overexpression 293A cells. **(C)** Representative mRNA profile of MEF2C in MEF2C-WT and MEF2C-MUT myoblasts at indicated time points after actinomycin D treatment. **(D,E)** MEF2C protein levels after YTHDF1 knockdown and overexpression on the 4th day after differentiation. *p < 0.05, **p < 0.01, siNC vs. siYTHDF1 samples; ^#^*p* < 0.05, empty Vector vs. OE-YTHDF1 samples, using Student's *t*-test. **(F,G)** MEF2C, YTHDF1, MYH3, GFP, and GAPDH protein levels in MEF2C-WT or MEF2C-MUT with YTHDF1 expression knocked down after myogenic induction for 4 days. **(H)** Myotube formation in MEF2C-WT or MEF2C-MUT with YTHDF1 expression knocked down after myogenic induction for 4 days (scale bar: 200 μm). **(I)** The fusion index was counted. **(J)** Relative dual-luciferase reporter activity of MEF2C-WT or MEF2C-MUT reporters in YTHDF1 overexpression 293A cells. **(K)** RIP-qPCR detection of the binding of YTHDF1 to the transcript of MEF2C in myoblasts transfected with the empty vector and MEF2C-WT. The results are presented as the means ± SD. ***p < 0.001, using Student's *t*-test. **(A–C,G,I,J)** *p < 0.05, **p < 0.01, ***p < 0.001, ns, no significance, using one-way ANOVA with Tukey's correction for multiple comparisons.

Our results have shown that METTL3 could promote the protein expression of MEF2C (Figures 4C,D). Therefore, it is plausible to hypothesize that METTL3, an m^6^A methyltransferase, could catalyze the methylation of *MEF2C* mRNA due to the increased m^6^A level of *MEF2C* in DM after myogenic differentiation (Figure 2E). Similarly, in the luciferase assay, we replaced *N*^6^-methylated adenosine with cytosine to synthesize a mutant *MEF2C*, and we ligated this sequence into the psiCHECK-2 dual-fluorescence vector. The relative luciferase activities of 293A cells transfected with the mutant *MEF2C* reporter were not significantly different when the cells were co-transfected with siMETTL3 or OE-METTL3. However, with the condition of the existence of the wild-type *MEF2C*-fused reporter, METTL3 knockdown and overexpression resulted in decreased and increased luciferase activity, respectively (Figures 5A,B). Thus, METTL3 was considered to regulate the m^6^A level of *MEF2C*.

As the most widely studied m^6^A readers, the m^6^A-dependent functions of the members of the YTH protein family include, but are not limited to, the regulation of mRNA translation efficiency, stability and splicing (17). There was no difference in the half-life of *MEF2C* mRNA between the myoblasts in the *MEF2C*-WT and *MEF2C*-MUT groups at 0, 3, 6, and 9 h after actinomycin D treatment (Figure 5C), indicating that YTHDF2 did not regulate the stability of *MEF2C* mRNA. Moreover, YTHDF1 has been recognized to enhance the translation of m^6^A-modified mRNAs and promote gene expression without changing the mRNA levels (22). To investigate whether MEF2C was modulated by YTHDF1, we examined the mRNA and protein levels of MEF2C after YTHDF1 knockdown and overexpression (Supplementary Figures 3A,B). Consistent with the reported functions of YTHDF1, overexpressing YTHDF1 improved the protein expression of MEF2C without affecting its mRNA level in myoblasts 4 days after the induction of differentiation. However, both the protein and mRNA levels of MEF2C were reduced after YTHDF1 expression was knocked down (Figures 5D,E, Supplementary Figures 3C,D). To further determine whether YTHDF1 functions as a direct m^6^A reader of *MEF2C*, Western blotting was performed, and the results showed that the expression of MEF2C was significantly decreased after YTHDF1 expression was knocked down in myoblasts expressing *MEF2C*-WT, whereas no significant change was observed in the myoblasts expressing *MEF2C*-MUT (Figures 5F,G). Moreover, knockdown of YTHDF1 in the *MEF2C*-MUT group could reversed the inhibition of MYH3 observed in the *MEF2C*-WT group (Figures 5F,G). The results of immunofluorescence staining of myotubes with a MYHC antibody were also consistent with the protein expression of MEF2C in the 4 groups. Among these groups, the myotube formation in the *MEF2C*-WT single treatment group was the most obvious, while the formation of myotubes was significantly inhibited after YTHDF1 expression was knocked down or in the *MEF2C*-MUT group (Figures 5H,I). Next, YTHDF1 overexpression resulted in increased luciferase activity in the presence of the wild-type *MEF2C*-fused reporter (Figure 5J). RIP using an antibody against YTHDF1 and anti-IgG followed by qPCR revealed that the m^6^A motif region in the *MEF2C* mRNA was effectively immunoprecipitated from bovine myoblasts overexpressing YTHDF1 (Figure 5K, Supplementary Figure 3E). These results indicated that YTHDF1 directly binds to *MEF2C* mRNA by recognizing the m^6^A modification site to promote MEF2C expression at the translational level.

Some studies have reported that METTL3 regulated myogenesis (28, 33, 46). Our results shown that METTL3 markedly enhanced the differentiation of bovine skeletal myoblasts by promoting the expression of some key myogenic factors (such as *MYOD1, MYOG, MYH3*, and *MYMK*) (Supplementary Figures 4A–D). After knocking down METTL3 expression, the formation of multinuclear myotubes was also significantly inhibited (as assessed by the fluorescence of MYHC protein) (Supplementary Figures 4E,F). The results suggested that METTL3 may regulate myogenic differentiation by promoting the expression of MEF2C in an m^6^A-YTHDF1-dependent manner. In general, these results indicate that METTL3 may increase the MEF2C protein levels by m^6^A-YTHDF1-dependent mRNA translation.

### MEF2C Promotes METTL3 Expression by Direct Binding Promoter DNA

**Figure 6:**
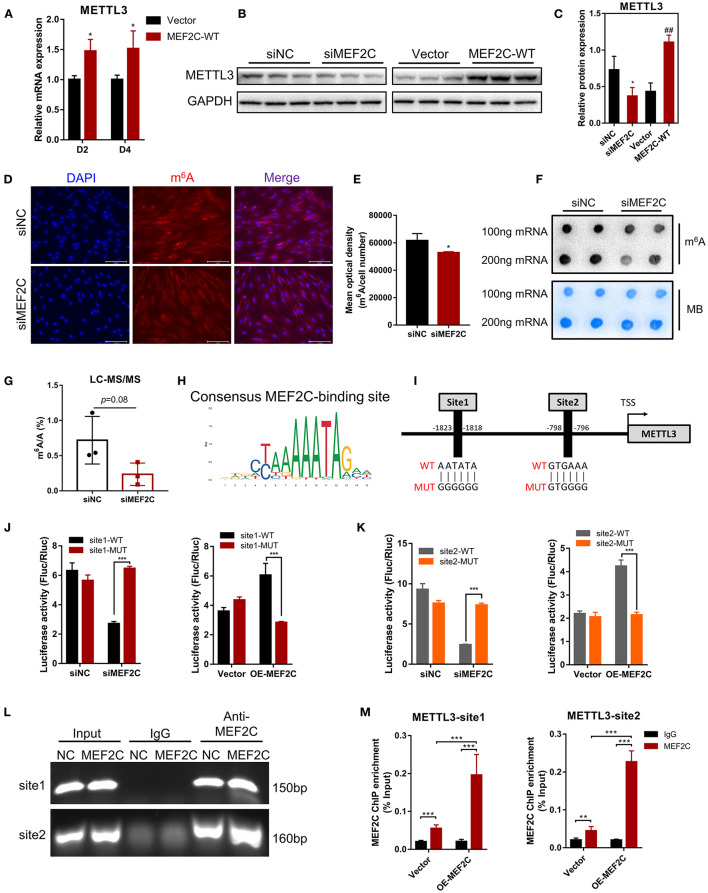
Protein expression of METTL3 is regulated by MEF2C via direct DNA-binding. **(A)** Overexpression of MEF2C promotes the expression of METTL3 mRNA on the 2nd and 4th days after differentiation. **(B,C)** METTL3 protein levels after MEF2C knockdown and overexpression on the 4th day after differentiation. *p < 0.05, **p < 0.01, siNC vs. siMEF2C samples; ^##^*p* < 0.01, empty Vector vs. MEF2C-WT samples; Student's *t*-test. **(D)** Knockdown of MEF2C expression decreased the m^6^A level of total RNA in bovine skeletal myoblasts after siRNA transfection for 3 days (scale bar: 100 μm). **(E)** Using Celleste Image Analysis Software (Invitrogen, USA), the average optical density was calculated as the cumulative optical density of m^6^A red fluorescence divided by the number of nuclei, *n* = 3. **(F,G)** Dot blot and LC-MS/MS assays showed the level of m^6^A after siMEF2C for 3 days. **(H,I)** The consensus MEF2C-binding site is indicated, and a schematic diagram illustrates two potential MEF2C-binding sites in the METTL3 promoter and the corresponding mutant sequences. **(J,K)** Luciferase assay with the wild-type or mutant sequences of METTL3 binding sites verified the activity of sites 1 and 2 in MEF2C knockdown or overexpression myoblasts. **(L)** ChIP and PCR analyses of myoblasts revealed the binding of MEF2C to two sites in the METTL3 promoter. **(M)** ChIP-qPCR detection of the binding of MEF2C to the 2 sites in the METTL3 promoter in myoblasts transfected with the empty vector and MEF2C-WT. **(A,E,G,J,K,M)** The results are presented as the means ± SD. *p < 0.05, **p < 0.01, ***p < 0.001, using Student's *t*-test.

As shown in Figure 6A, *METTL3* mRNA expression was enhanced on the 2nd and 4th days after the induction of differentiation in MEF2C overexpressing myoblasts. Western blotting showed that a significant decrease or increase in the METTL3 protein levels was observed in the MEF2C knockdown or overexpression cells, respectively (Figures 6B,C). Immunofluorescence staining revealed that knockdown of MEF2C expression reduced the m^6^A levels in the total RNA of bovine myoblasts (Figures 6D,E). Meanwhile, Dot blot and LC-MS/MS showed that m^6^A levels were decreased on day 3 of myogenic differentiation in siMEF2C myoblasts (Figures 6F,G).

MEF2C is a transcription factor that promotes the transcription of genes by recognizing and binding to transcription factor-binding sites (TFBS) of downstream target genes (44, 47). Two potential MEF2C-binding sites in the *METTL3* promoter were identified by genomic analysis and predicted with the JASPAR (48) and AnimalTFDB (49) online tools, and these results revealed the mechanisms underlying function of MEF2C (Figures 6H,I). Then, we designed two pairs of primers covering the two MEF2C-binding sites (Figure 6I) and performed a luciferase assay in 293A cells transfected with siMEF2C or the MEF2C expression vector (OE-MEF2C). MEF2C expression increased the luciferase activities of cells expressing both wild-type promoters, while these effects were eliminated by a mutation in site 1 or site 2 (Figures 6J,K). To further confirm whether MEF2C can directly bind to the *METTL3* promoter, ChIP assays were carried out. As shown in Figures 6L,M the ChIP-qPCR results demonstrated that MEF2C bound to both sites. Collectively, these results provide evidence that MEF2C directly binds to the *METTL3* promoter as a transcription factor to increase METTL3 expression.

## Discussion

**Figure 7:**
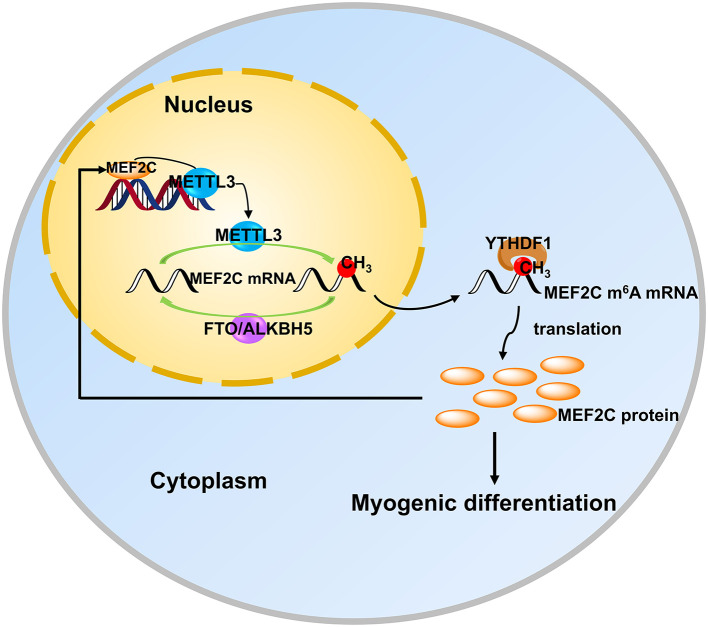
MEF2C promotes the differentiation of bovine myoblasts by posttranscriptional activation of the METTL3-m^6^A-YTHDF1 axis. METTL3 catalyzes the *N*^6^-methylation of MEF2C mRNA, and YTHDF1 recognizes this modification site to promote the translation of MEF2C. Then, MEF2C can directly bind to the promoter region of METTL3 to activate its expression, suggesting that there is a positive feedback loop underlying the process of bovine skeletal myoblast differentiation.

In recent years, the two extraordinary discoveries, i.e., the methylation modification of RNA is as reversible as DNA methylation (15) and the establishment of m^6^A-specific methylated RNA immunoprecipitation coupled with next-generation sequencing (MeRIP-seq) (39, 50), have accelerated advances in research on m^6^A modification in various fields. Meanwhile, a series of mechanisms in myogenic differentiation are also being uncovered uninterruptedly. However, the potential and detailed molecular mechanism underlying m^6^A modification in myogenic differentiation is largely unknown. In the present study, we found that the level of m^6^A decreased after myogenic differentiation in bovine skeletal myoblast, which was consistent with the results in C2C12 cells and sheep myoblasts (33, 34). To investigate the molecular mechanisms by which m^6^A methylation influences myogenic differentiation, we further analyzed our previous sequencing data and found that the mRNA expression of MEF2C was up-regulated in myogenic differentiation, while the m^6^A level was also significantly up-regulated. Further experiments demonstrated that MEF2C is regulated by METTL3-m^6^A-YTHDF1 axis and METTL3 may promote myogenic differentiation by mediating the expression of MEF2C. In turn, MEF2C directly binds to the *METTL3* promoter, promoting *METTL3* transcription and thus affecting m^6^A levels (Figure 7).

MEF2C, the member of the MEF2 family that is expressed first in skeletal muscle development, plays a positive role in skeletal muscle differentiation and regeneration (44, 45, 47, 51). We verified that MEF2C promoted the myogenic differentiation of bovine skeletal myoblasts by knockdown and overexpression assays, which was consistent with the findings of previous reports (44, 47). Furthermore, through synonymous mutation of the m^6^A motif in the MEF2C coding region, we found that the m^6^A modification was necessary for MEF2C protein expression and myotube formation. Then, we performed loss-of-function and gain-of-function assays to analyze the effects of active m^6^A methyltransferase (METTL3) and demethylase (FTO), respectively, and found that the effects of METTL3 and FTO on the protein expression of MEF2C were opposite of those RNA methylation modification. Similarly, the protein expression of MEF2C was also affected by a methylation inhibitor (DAA). Multiple lines of evidence indicated that the protein expression of MEF2C was positively correlated with the level of m^6^A. We then found that METTL3 regulates MEF2C expression by mediating m^6^A methylation in myoblast differentiation. Some studies have found that METTL3 not only acts as m^6^A writer, but also recognizes and binds m^6^A sites to promote protein translation, and yet this generally occurs in the 3'UTR of the target transcripts (17), whereas the differentially methylated peaks of *MEF2C* are positioned in the CDS. Therefore, we speculated that YTHDF1, an m^6^A reader protein, may be responsible for the increased expression of MEF2C. Our results demonstrated that YTHDF1 promoted the protein expression of MEF2C, and RIP assays indicated that YTHDF1 directly bound to *MEF2C* mRNA. Thus, multiple lines of evidence support our hypothesis that the *MEF2C* transcript was directly targeted by YTHDF1 and that METTL3 leads to high protein expression of MEF2C in an m^6^A-YTHDF1-dependent manner in myoblast differentiation. Consistent with our findings, the previously reported next-generation sequencing data of m^6^A, CLIP and RIP from the m6A2Target Database (http://m6a2target.canceromics.org/#/search/MEF2C) also identified *MEF2C* mRNA as a potential target of m^6^A writers, erasers, and readers in humans and mice (GSE46705, GSE79577, GSE56010, GSE90914, GSE102493, GSE94098, GSE94148, and GSE100528). Furthermore, we aligned the m^6^A modified sequence of bovine MEF2C mRNA with that of other species (including humans, mice, pigs and sheep) and found that the coding region and m^6^A motif sequence of *MEF2C* were very conserved (Supplementary Figure 5). These findings suggest a possible general regulatory role of m^6^A modifications in muscle development. Besides, we found a consequent alteration in METTL3 expression while conducting MEF2C knockdown and overexpression assays. As a well-known transcription activator, MEF2C was found to facilitate the expression of METTL3 by directly binding to the *METTL3* promoter during myogenic differentiation in our study.

Strikingly, the m^6^A demethylase FTO also affected the expression of MEF2C, and two protein bands of similar size appeared in MEF2C at day 4 of myogenic differentiation after knockdown of FTO (Figure 4A). Previous studies have found that m^6^A demethylase FTO can regulate the splicing of the target transcripts (52, 53), so we speculated that FTO may regulate the translation of two different transcripts of *MEF2C*, which needs to be confirmed by subsequent studies. It could also be a protein modification that changes the weight of the protein.

Uniquely, our results showed that both m^6^A methyltransferase (METTL3) and m^6^A demethylases (FTO and ALKBH5) exhibited increased expression after the onset of differentiation. Simultaneously, overall m^6^A levels were reduced, but METTL3 promoted myogenic differentiation of bovine myoblasts. These seemingly contradictory results imply that m^6^A methylation modifications in skeletal myogenesis are dynamically changing and there may be a complex regulatory network of m^6^A methylation. The combined analysis of MeRIP-seq and RNA-seq data showed no significant correlation between differential gene expression and differential m^6^A abundance (Figure 2A), which is consistent with the *N*^6^-methyladenosine methylome profile in porcine muscle development (54), whereas the m^6^A modification profiles of goose embryonic muscle shown that m^6^A methylation was negatively correlated with transcript level (55). These results indicate that the regulation of mRNA transcription by m^6^A modification during myogenic differentiation was complicated.

Similarly, METTL3 is essential for *MyoD* mRNA expression in proliferating C2C12 myoblasts for skeletal muscle differentiation (28), is required for skeletal muscle regeneration (56) in mice and regulates the transitions of muscle stem cells/myoblasts (33). A recent study shown that METTL3 regulates skeletal muscle specific miRNAs at both the transcriptional and posttranscriptional levels (46). As suggested by these previous studies, METTL3 plays a critical role in skeletal muscle homeostasis and myogenic differentiation as a result of its function as a methyltransferase. However, some investigators identified that METTL3 inhibited differentiation of the C2C12 cells and primary mouse skeletal muscle cells (33, 35, 56). We analyzed the reasons for these differential results and found an intriguing result. Apart from the difference in the origin and type of cells, the different media used in cell culture are likely to be responsible for the opposite results of METTL3 regulation of myogenic differentiation. METTL3 promoted myogenic differentiation of cells cultured using growth medium with 20% FBS, including C2C12 cells (28) and the primary bovine skeletal myoblasts we used. The opposite result was obtained for studies using 10% or 15% FBS, i.e., METTL3 inhibited myogenic differentiation of C2C12 cells or primary mouse skeletal myoblasts (33, 35, 56). It is also important to mention that the differentiation medium used in all these studies during myogenic differentiation of cells was 2% horse serum. These results implies that there is still a lot of work to be explored and developed in the field of RNA methylation.

In conclusion, we found that the protein expression of MEF2C was positively regulated by the METTL3-m^6^A-YTHDF1 axis in myoblast differentiation. In addition, MEF2C promoted the expression of METTL3 by binding to its promoter, thus there may be a positive feedback loop between these molecules in myoblast differentiation (Figure 7). Our findings could provide new Q15insights into skeletal muscle development and livestock breeding. Moreover, it is becoming increasingly clear that the mechanisms of RNA methylation in skeletal myogenesis.

## Data Availability Statement

The original contributions presented in the study are included in the article/Supplementary Material, further inquiries can be directed to the corresponding author/s.

## Author Contributions

XY and LZ designed the experiment. XY performed most experiments, analyzed the data, and wrote the manuscript. YN provided bovine primary myoblasts and conducted partial cellular experiments. SA conducted partial molecular experiments. CM contributed to the discussion. All authors contributed to the article, provided constructive suggestions for manuscript writing, and approved the submitted version.

## Funding

This work was supported by the National Key Research and Development Program of China (2018YFD0501700), National Natural Science Foundation of China (31972994), Key Research and Development Program of Ningxia Province (2019BEF02004), National Beef and Yak Industrial Technology System (CARS-37), and Key Research and Development Program of Shaanxi Province (2022NY-050 and 2022ZDLNY01-01).

## Conflict of Interest

The authors declare that the research was conducted in the absence of any commercial or financial relationships that could be construed as a potential conflict of interest.

## Publisher's Note

All claims expressed in this article are solely those of the authors and do not necessarily represent those of their affiliated organizations, or those of the publisher, the editors and the reviewers. Any product that may be evaluated in this article, or claim that may be made by its manufacturer, is not guaranteed or endorsed by the publisher.
