# Supplementary material for: MEF2C Expression Is Regulated by the Post-transcriptional Activation of the METTL3-m6A-YTHDF1 Axis in Myoblast Differentiation
Source: Front Vet Sci. 2022 Apr 28;9:900924. doi: 10.3389/fvets.2022.900924 (PMC9096896; doi:10.3389/fvets.2022.900924)
Supplement: Supplementary file 1 [file Table_1.DOCX]

Table S1. Primers used in this study

| **Primer name** | **Sequence (5’-3’)** |
| --- | --- |
| **Primers for RT-qPCR** | |
| qGAPDH-F | AGTTCAACGGCACAGTCAAGG |
| qGAPDH-R | ACCACATACTCAGCACCAGCA |
| qMEF2C-F | CCTGATGCAGACGATTCAGTAG |
| qMEF2C-R | AAAGTTGGGAGGTGGAACAG |
| qMYOD1-F | AACCCCAACCCGATTTACC |
| qMYOD1-R | CACAACAGTTCCTTCGCCTCT |
| qMYOG-F | GGCGTGTAAGGTGTGTAAG |
| qMYOG-R | CTTCTTGAGTCTGCGCTTCT |
| qMYH3-F | TGAACGCCCTCTCCAAATCC |
| qMYH3-R | AATGAAGTGCTGTCTCGGCA |
| qMYMK-F | TCGGCCATCCTCATCATTG |
| qMYMK-R | CGTACGTGTAGTCCCAGTCCTC |
| qMETTL3-F | TCGAAAGCTGCACTTCAGAC |
| qMETTL3-R | TCCAACGCTCTGTGTAAGGG |
| qFTO-F | AGCAGCGTACAACGTCACTT |
| qFTO-R | AGGGTCGTCCTCACTTTCCT |
| qALKBH5-F | TACTTCTTCGGCGAGGGCTA |
| qALKBH5-R | TGGTAGTCGTTGATGACGGC |
| qYTHDF1-F | CAGGCATCCTTTCTGGCAAC |
| qYTHDF1-R | AGGTCCCTTGTTGTCCCAAG |
| **Primers for plasmid construction** | |
| FTO-cds-F | ctagctagcATGAAGCGGACCCCGACG |
| FTO-cds-R | ccgctcgagCTAGGGCCTGGTTTCCAG |
| METTL3-cds-F | ctagctagcATGTCGGACACGTGGAGC |
| METTL3-cds-R | cggggtaccCTATAGATTCTTAGGTTTAGAG |
| YTHDF1-cds-F | cgcggatccATGTCGGCCACCAGCGTG |
| YTHDF1-cds-R | ccggaattcCCCTCACTGCTTGTTTCG |
| MEF2C-cds-F | ctagctagcATGGGGAGAAAAAAGATTC |
| MEF2C-cds-R | cgcggatccTCATGTTGCCCATCCTT |
| **Primers for MeRIP-qPCR and RIP-qPCR** | |
| MEF2C-m^6^A-F | TCACCGGAACGAATTCCACT |
| MEF2C-m^6^A-R | GCCCATCCTTCAGAGAGTCG |
| **Primers for ChIP-qPCR** | |
| METTL3-site1-F | AAATGTCCCCTGTTGTATTCTGA |
| METTL3-site1-R | GTGTATGCCATGTTGAACCAGA |
| METTL3-site2-F | GAAAAGACCCTGATGCT |
| METTL3-site2-R | AAATGGTAACCCACTCC |
