# Supplementary material for: MEF2C Expression Is Regulated by the Post-transcriptional Activation of the METTL3-m6A-YTHDF1 Axis in Myoblast Differentiation
Source: Front Vet Sci. 2022 Apr 28;9:900924. doi: 10.3389/fvets.2022.900924 (PMC9096896; doi:10.3389/fvets.2022.900924)
Supplement: Supplementary file 2 [file Table_2.DOCX]

Table S2. siRNAs used in this study

| **siRNA name** | **Sequence (5’-3’)** |
| --- | --- |
| siMEF2C-1-F | GCUGUGUGACUGUGAGAUUTT |
| siMEF2C-1-R | AAUCUCACAGUCACACAGCTT |
| siMEF2C-2-F | CAGGCAGCAAGAAUACAAUTT |
| siMEF2C-2-R | AUUGUAUUCUUGCUGCCUGTT |
| siMEF2C-3-F | CCAGGCAGCAAGAAUACAATT |
| siMEF2C-3-R | UUGUAUUCUUGCUGCCUGGTT |
| siFTO-1-F | CCAUAAAGAGGUUCAACAATT |
| siFTO-1-R | UUGUUGAACCUCUUUAUGGTT |
| siFTO-2-F | GCUGUGCUUCGCGAAGUUATT |
| siFTO-2-R | UAACUUCGCGAAGCACAGCTT |
| siMETTL3-1-F | GCACUUGGAUCUUCGGAAUTT |
| siMETTL3-1-R | AUUCCGAAGAUCCAAGUGCTT |
| siMETTL3-2-F | CCACAUGGAUACCUGCAAATT |
| siMETTL3-2-R | UUUGCAGGUAUCCAUGUGGTT |
| siYTHDF1-1-F | GGCGUGUGUUCAUCAUCAATT |
| siYTHDF1-1-R | UUGAUGAUGAACACACGCCTT |
| siYTHDF1-2-F | GCACAGCACCUCCAUCUUUTT |
| siYTHDF1-2-R | AAAGAUGGAGGUGCUGUGCTT |
| siYTHDF1-3-F | CCUCCAUCUUUGACGACUUTT |
| siYTHDF1-3-R | AAGUCGUCAAAGAUGGAGGTT |
