# Supplementary material for: MEF2C Expression Is Regulated by the Post-transcriptional Activation of the METTL3-m6A-YTHDF1 Axis in Myoblast Differentiation
Source: Front Vet Sci. 2022 Apr 28;9:900924. doi: 10.3389/fvets.2022.900924 (PMC9096896; doi:10.3389/fvets.2022.900924)
Supplement: Supplementary file 3 [file Table_3.DOCX]

Table S3. Antibodies used in this study

| **Antibodies** | **Indentifier and Source** |
| --- | --- |
| anti-m^6^A | 202003, Synaptic Systems |
| anti-GAPDH | ab181603, Abcam |
| anti-MYH3 | sc-53091, Santa Cruz |
| anti-MYOG | ab264517, Abcam |
| anti-MEF2C | 10056-1-AP, Proteintech |
| anti-GFP Tag | 50430-2-AP, Proteintech |
| anti-METTL3 | ab195352, Abcam |
| anti-FTO | ab126605, Abcam |
| anti-YTHDF1 | 17479-1-AP, Proteintech |
| anti-PAX7 | ab187339, Abcam |
| anti-MyoD1 | ab16148, Abcam |
| anti-MYHC | GTX20015, GeneTex |
| HRP-conjugated goat anti-mouse IgG | D110087, Sangon Biotech |
| HRP-conjugated goat anti-rabbit IgG | D110058, Sangon Biotech |
| Alexa Fluor 555-conjugated donkey anti-rabbit IgG | ab150074, Abcam |
| Alexa Fluor 488-conjugated goat anti-mouse IgG | ab150113, Abcam |
| Alexa Fluor 555-conjugated donkey anti-mouse IgG | ab150106, Abcam |
