# Supplementary figures and images for: MEF2C Expression Is Regulated by the Post-transcriptional Activation of the METTL3-m6A-YTHDF1 Axis in Myoblast Differentiation
Source: Front Vet Sci. 2022 Apr 28;9:900924. doi: 10.3389/fvets.2022.900924 (PMC9096896; doi:10.3389/fvets.2022.900924)

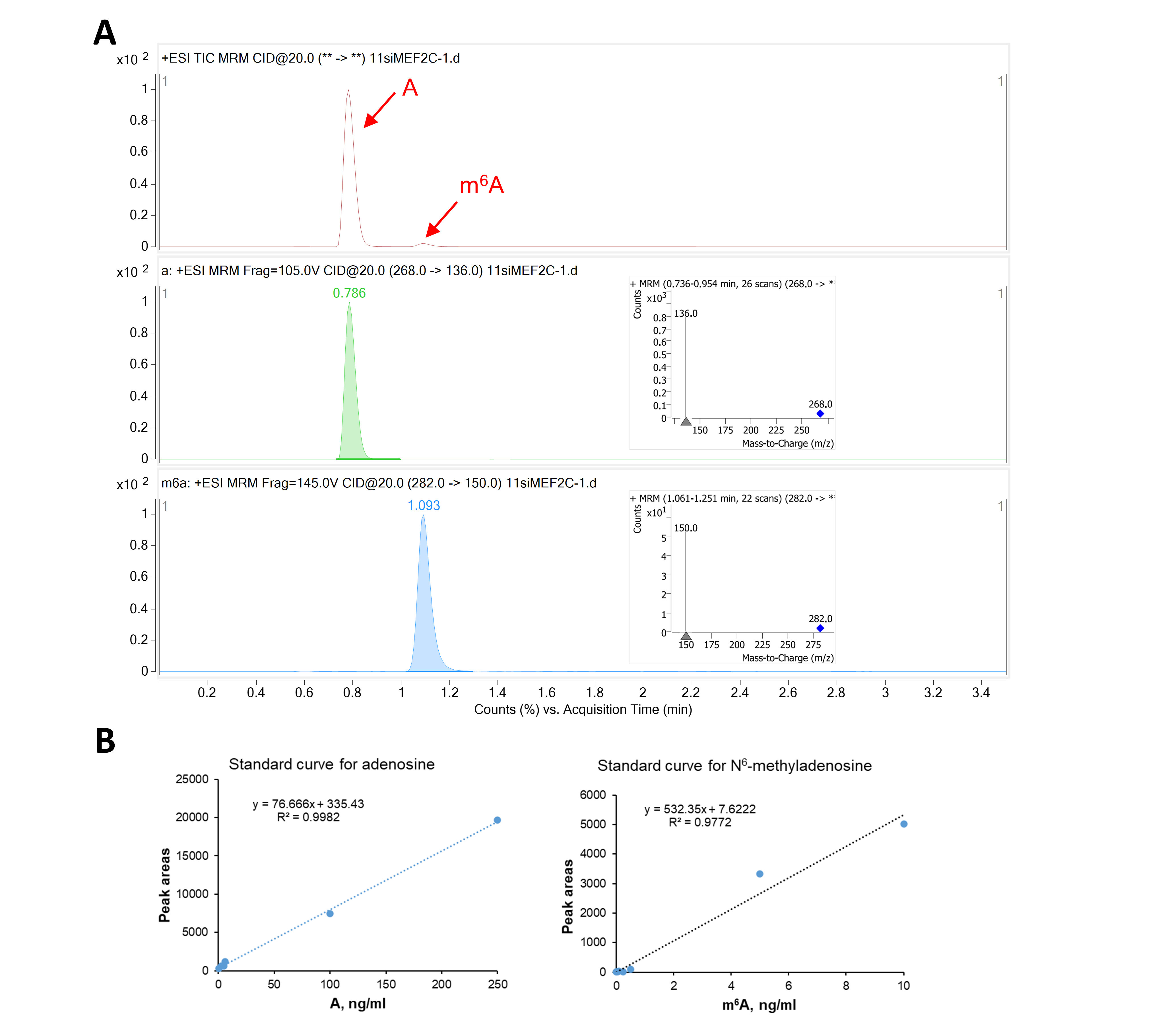

Supplement: Supplementary file 6 [file Image_1.TIF]

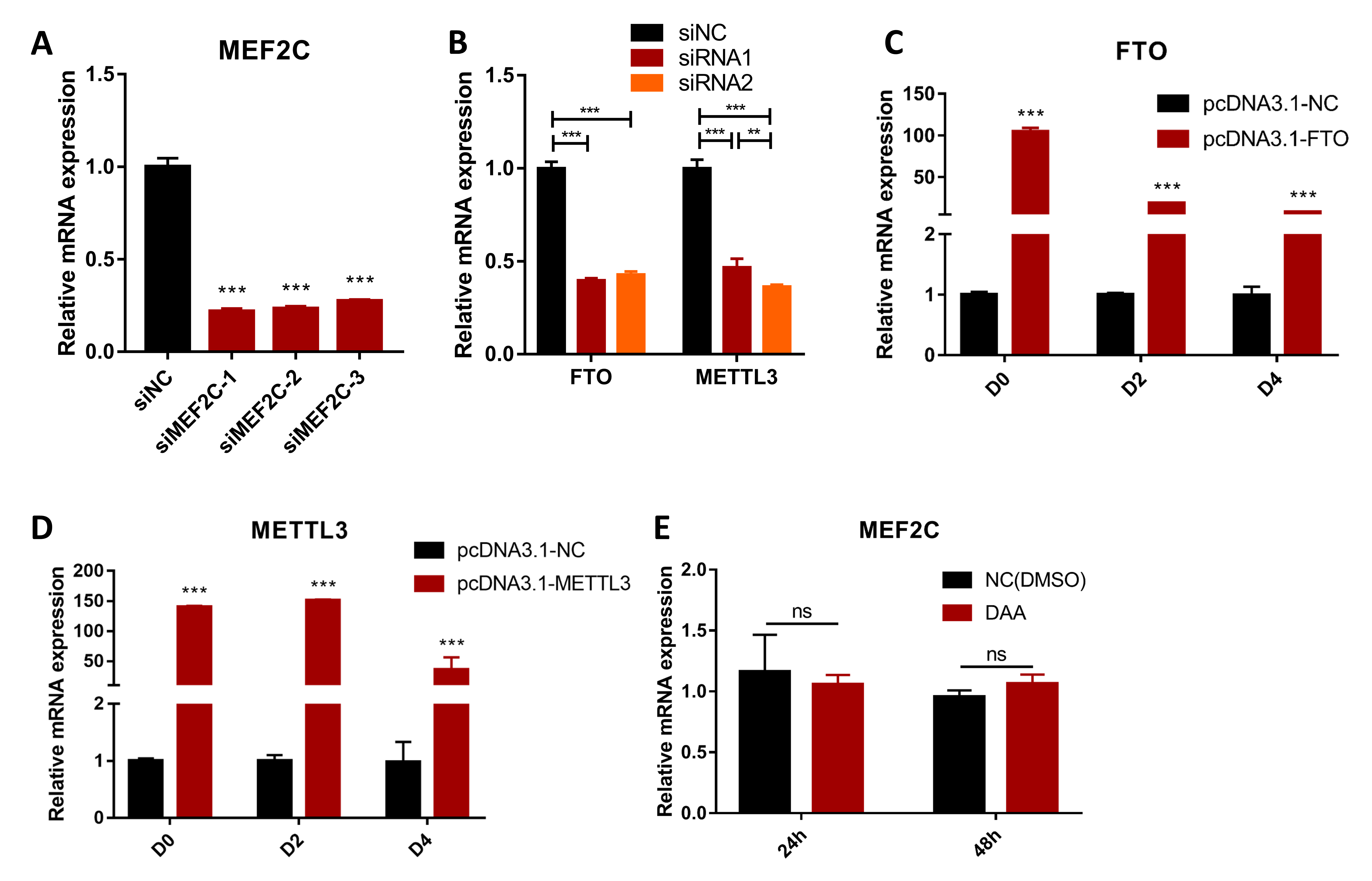

Supplement: Supplementary file 7 [file Image_2.TIF]

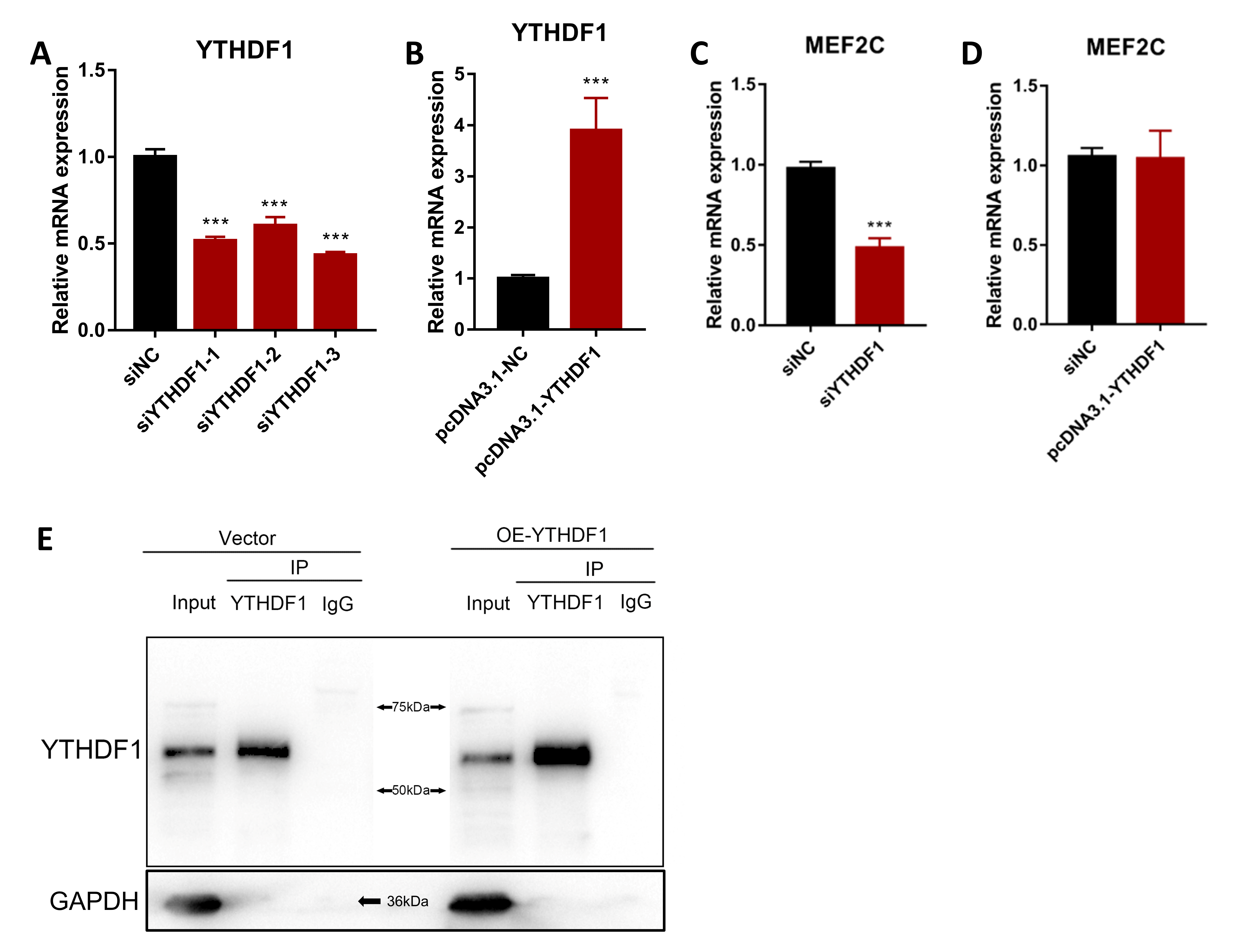

Supplement: Supplementary file 8 [file Image_3.TIF]

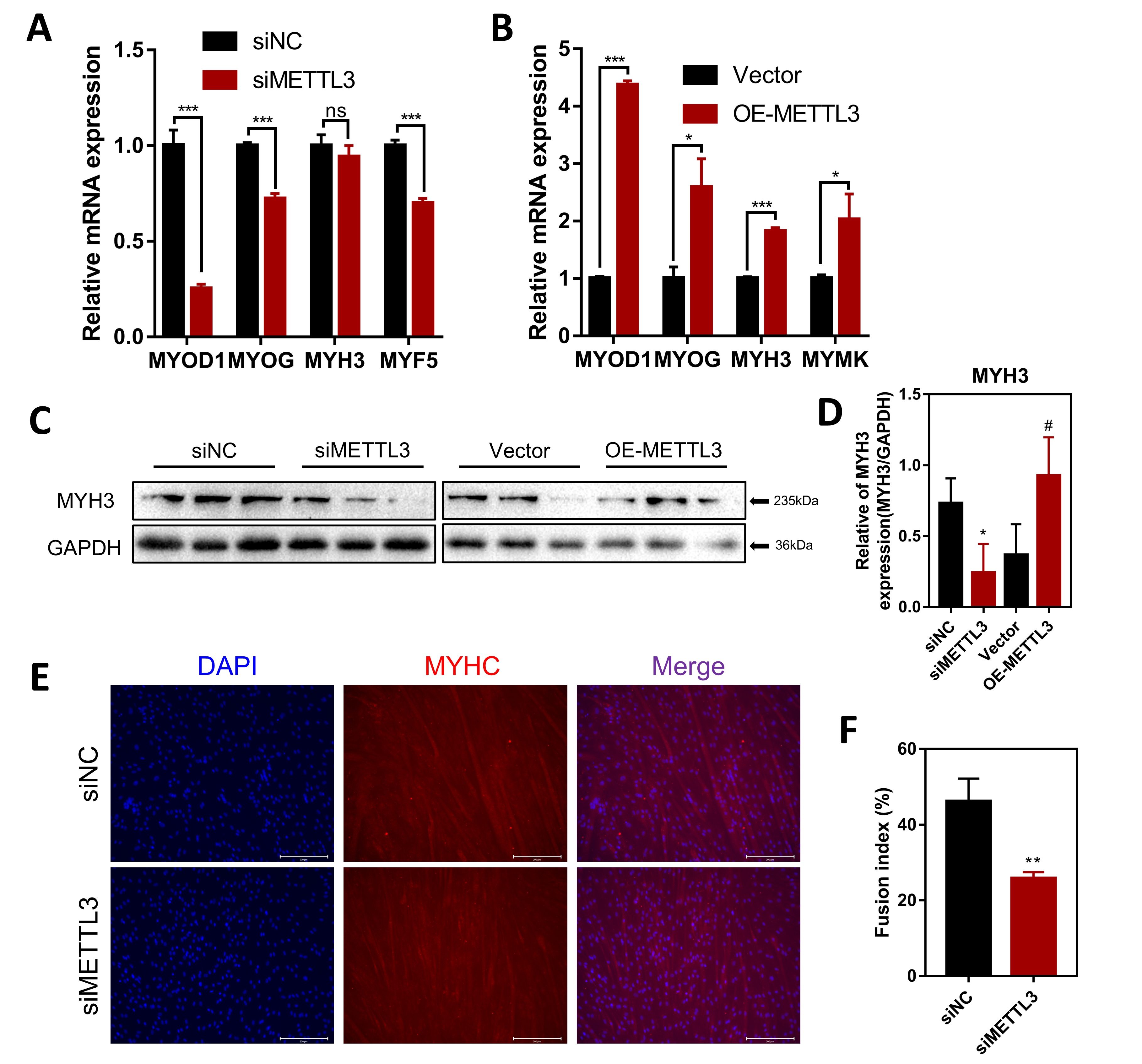

Supplement: Supplementary file 9 [file Image_4.TIF]

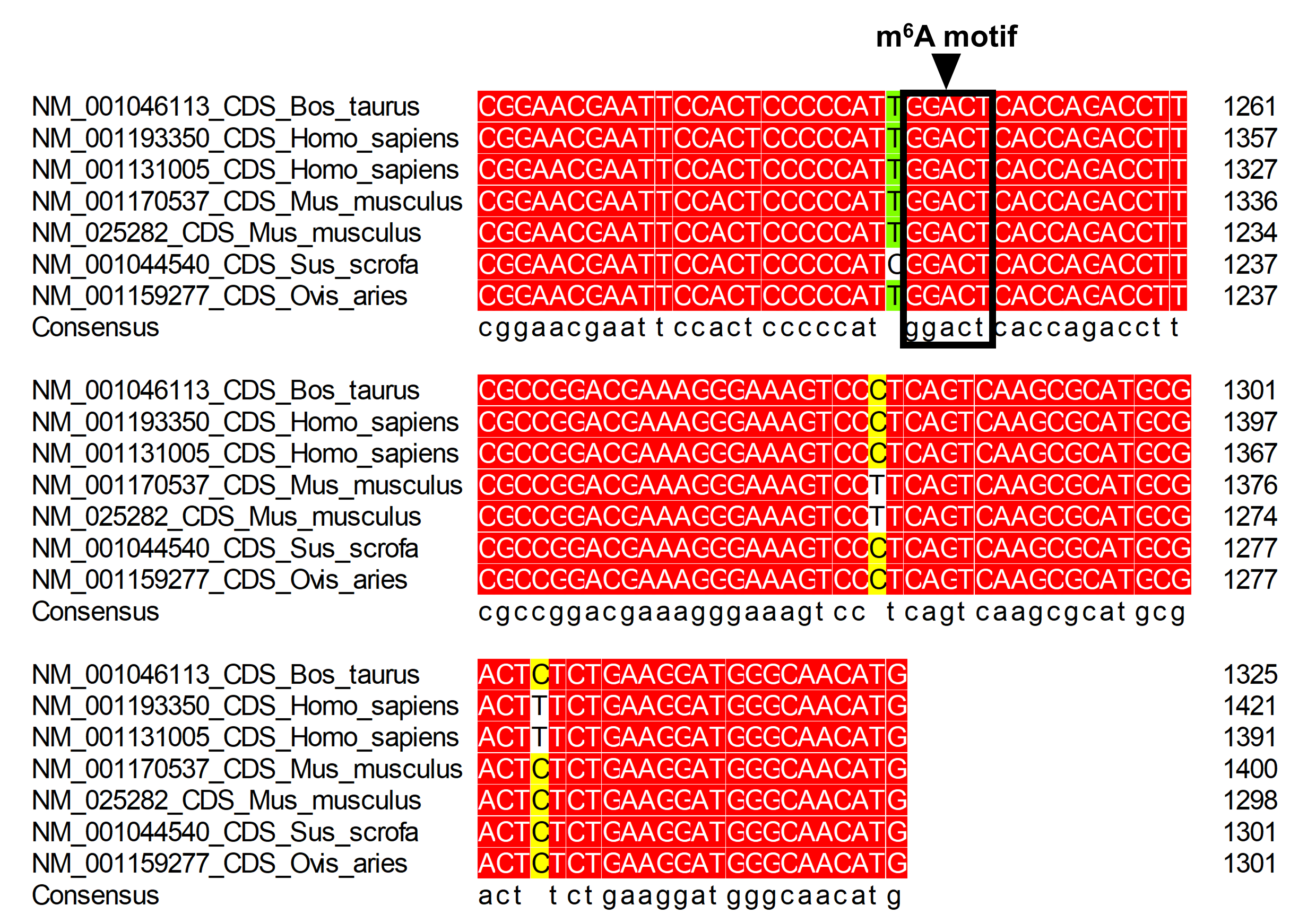

Supplement: Supplementary file 10 [file Image_5.TIF]
